# Supplementary material for: Prediction of SARS-CoV-2 transmission dynamics based on population-level cycle threshold values: An epidemic transmission and machine learning modeling study
Source: eLife. 2026 Feb 16;15:e95666. doi: 10.7554/eLife.95666 (PMC13155751; doi:10.7554/eLife.95666)
Supplement: Supplementary file 1. [file elife-95666-supp1.docx]

| **Vaccination Phase*** | **Wave** | **VoC** | **Target population for vaccination** |
| --- | --- | --- | --- |
| Phase 1 | Waves 1, 2, 3  Dec 14 2020 - Mar 7 2021 | Wildtype SARS-CoV-2 | Residents, staff and essential visitors to long-term care settings; individuals assessed and awaiting a long-term care placement; health care workers providing care for COVID-19 patients; and remote and isolated Indigenous communities. |
| Phase 2 | Wave 3  Mar 8 2021 -  Apr 2021 | Alpha (B.1.1.7)  Gamma (P.1) | Individuals age ≥80; Indigenous peoples age ≥65 and Indigenous Elders; Indigenous communities; hospital staff, community general practitioners and medical specialists; vulnerable populations in select congregate settings; and staff in community home support and nursing services for seniors. |
| Phase 3 | Wave 3  Apr 15 2021 - May 10 2021 | Alpha (B.1.1.7)  Gamma (P.1) | Individuals aged 60-79 years, Indigenous peoples aged 18-64 and people aged 16-74 who are clinically extremely vulnerable. |
| Phase 4 | Waves 3, 4  May 11 2021- Jul 17 2021 | Alpha (B.1.1.7)  Gamma (P.1) | Everyone aged ≥12 years-old. From September 2021, third vaccine dose available for people who are clinically extremely vulnerable |
| Phase 4 | Wave 4  Jul 18 2021 - Nov 18 2021 | Delta (B.1.617.2) |  |
| Phase 5 | Wave 4  Nov 19 2021 - Jan 8 2022 | Omicron | Everyone aged ≥5 years-old. From the end of November 2021, children aged 5-11 are eligible for vaccination. Everyone aged ≥18 and invited to get a ‘booster’ (third vaccine dose) within 6-8 months after receipt of their second dose. |

*Vaccination phases were defined by vaccine eligibility of the target populations in BC, and are detailed separately (12)

SARS-CoV-2: SARS-CoV-2: severe acute respiratory syndrome coronavirus type 2
