# Supplementary material for: Prediction of SARS-CoV-2 transmission dynamics based on population-level cycle threshold values: An epidemic transmission and machine learning modeling study
Source: eLife. 2026 Feb 16;15:e95666. doi: 10.7554/eLife.95666 (PMC13155751; doi:10.7554/eLife.95666)
Supplement: Supplementary file 2. [file elife-95666-supp2.docx]

**Supplementary File 2.** Epidemiological, clinical and laboratory data of the earlier British Columbia SARS-CoV-2 pandemic phases

| **Group** | **Subgroup** | **Phase 1**  **(Dec 14 2020 - May 10 2021)** | **Phase 2.1**  **(May 11 2021 - July 17 2021)** | **Phase 2.2**  **(July 18 2021 - Nov 18 2021)** |
| --- | --- | --- | --- | --- |
| Testing | Positives | 96406 | 12079 | 68370 |
|  | Negatives | 1154936 | 282466 | 1003638 |
|  | Repeats | 41115 | 5550 | 36757 |
|  | Other | 5073 | 1034 | 3532 |
| Specimen type | NP | 59702 | 7457 | 33899 |
|  | SG | 24988 | 4475 | 34079 |
|  | Other | 11716 | 147 | 392 |
| No *E* gene Result | | 16239 | 2456 | 16516 |
| Age | 0-4 | 1918 | 300 | 1832 |
|  | 5-18 | 11656 | 1925 | 12580 |
|  | 19-39 | 40179 | 5017 | 27138 |
|  | 40-59 | 26376 | 3121 | 16992 |
|  | 60-79 | 12771 | 1421 | 7901 |
|  | 80+ | 3091 | 295 | 1918 |
|  | Unknown | 415 | 0 | 9 |
| Sex | Male | 49957 | 6336 | 34679 |
|  | Female | 45928 | 5659 | 32895 |
|  | Unknown | 521 | 84 | 796 |
| Patient health authority | 1 | 54245 | 6898 | 23429 |
|  | 2 | 8187 | 1839 | 18837 |
|  | 3 | 5946 | 462 | 9451 |
|  | 4 | 23528 | 2512 | 9182 |
|  | 5 | 3973 | 314 | 7161 |
|  | Unknown | 527 | 54 | 310 |
| Vaccination status | Unvaccinated | 94013 | 9887 | 43612 |
|  | One dose | 2239 | 2044 | 6317 |
|  | Fully vaccinated | 154 | 148 | 18367 |
|  | Other | 0 | 0 | 74 |
| VoC lineage | Alpha | 74 | 10 | 0 |
|  | Beta | 253 | 483 | 22220 |
|  | Delta | 4595 | 2134 | 99 |
|  | Gamma | 0 | 0 | 0 |
|  | Omicron | 293 | 39 | 307 |

NP: nasopharyngeal; SG: saline gargle; VoC: variant of concern
