# Supplementary material for: Prediction of SARS-CoV-2 transmission dynamics based on population-level cycle threshold values: An epidemic transmission and machine learning modeling study
Source: eLife. 2026 Feb 16;15:e95666. doi: 10.7554/eLife.95666 (PMC13155751; doi:10.7554/eLife.95666)
Supplement: Supplementary file 3. [file elife-95666-supp3.docx]

| **Type of assay** | **Assay used**  **(manufacturer)** | **Extraction** | **PCR** | **Ct Interpretation criteria** |
| --- | --- | --- | --- | --- |
| Laboratory-developed | BCCDC PHL LDT | MagMax | ABI 7500 | Ct threshold for positivity: 38 |
| Commercial | Xpert Xpress CoV-2 Plus (Cepheid) | | | Manufacturer recommended threshold |
|  | Panther Fusion (Hologic) | | |  |

BCCDC PHL: British Columbia Centre for Disease Control Public Health Laboratory; Ct: cycle threshold; LDT: laboratory-developed test; PCR: polymerase chain reaction; SARS-CoV-2: severe acute respiratory syndrome coronavirus type 2
