# Supplementary material for: Prediction of SARS-CoV-2 transmission dynamics based on population-level cycle threshold values: An epidemic transmission and machine learning modeling study
Source: eLife. 2026 Feb 16;15:e95666. doi: 10.7554/eLife.95666 (PMC13155751; doi:10.7554/eLife.95666)
Supplement: Supplementary file 4. [file elife-95666-supp4.docx]

| **Names*** | **Description** | **Fixed** | **Values** | **Priors** | **Lower**  **bound** | **Upper**  **bound** |
| --- | --- | --- | --- | --- | --- | --- |
| tshift | Time from infection to initial viral growth | yes | 0 | - | 0 | 3 |
| viral_peak | Modal Ct Value at peak viral load | no | 19.7359875 | Normal(19.7, 2.00) | 0 | 40 |
| obs_sd | Initial scale parameter for the Gumbel Distribution until a = t_eclipse + t_peak + t_switch | no | 5 | Normal(5.00, 0.50) | 0 | 25 |
| sd_mod | Multiplicative factor applied to scale parameter for the Gumbel distribution starting at a = teclipse+tpeak+tswitch+tscale | yes | 0.78 | - | 0 | 1 |
| sd_mod_wane | Time from secondary waning phase until Gumbel distribution reaches its minimum scale parameter | yes | 14 | - | 0 | 14 |
| true_0 | Ct value at time of infection | yes | 40 | - | 40 | 100 |
| intercept | Limit of detection of Ct value | yes | 40 | - | 35 | 100 |
| LOD | Limit of detection of viral load (log10 RNA copies / mL) | yes | 3 | - | 0 | 10 |
| incu | Time from initial viral growth to peak viral load | yes | 5 | - | 0 | 10 |
| t_switch | Time from peak viral load to secondary waning phase | no | 13.3 | Normal(13.3, 3.00) | 0 | 30 |
| level_switch | Modal Ct Value at a = t_eclipse + t_peak + t_switch | yes | 38 | - | 0 | 40 |
| wane_rate2 | Time from infection until modal Ct value is equal to the limit of detection | yes | 1000 | - | 0 | 10000 |
| prob_detect | Daily probability of detectability loss | no | 0.1032993 | Beta(10.5, 91.2) | 0 | 1 |
| R_0_ | Basic Reproductive Number | no | 2 | Log-normal(log(2.00), 0.60), and is bounded between Uniform(1.00, 10.0) | 1 | 10 |
| infectious* | Infectious period | yes | 3 | - | 0 | 25 |
| incubation* | Incubation period | yes | 4 | - | 0 | 25 |
| t_0_* | Effective seed time | yes | 1 | - | 0 | 100 |
| I_0_* | Proportion infected at seed time | no | 0.0001 | Normal (0.0001,0.01) | 0 | 0.01 |

*These parameters were modified compared to previously-published work (3) for the current study to account for Omicron variant kinetics as described in the Methods.

SEIR: susceptible-exposed-infected-recovered
