# Supplementary material for: Prediction of SARS-CoV-2 transmission dynamics based on population-level cycle threshold values: An epidemic transmission and machine learning modeling study
Source: eLife. 2026 Feb 16;15:e95666. doi: 10.7554/eLife.95666 (PMC13155751; doi:10.7554/eLife.95666)
Supplement: Supplementary file 5. [file elife-95666-supp5.docx]

| **Hyperparameter** | **Lasso** | **RF** | **LGBM** | **XGBoost** | **Catboost** |
| --- | --- | --- | --- | --- | --- |
| Alpha | [0.001,0.01,0.1] | - | - | - | - |
| Max Depth | - | [2,4,8,16,32] | [2,4,8] | [6,10] | - |
| Number of Estimators | - | [4,16,64,256] | - | - | - |
| Minimum Sample Split | - | [2,4,8,16,32] | - | - | - |
| Number of Leaves | - | - | [4,8,16,32,64,128] | - | - |
| Minimum Data in Leaf | - | - | [2,4,8,16,32] | - | - |
| Depth | - | - | - | - | [1,5,10] |
| Iterations | [500,1000,2000] | - | - | - | [250,500,1000] |
| Learning Rate | - | - | - | - | [0,001, 0.01, 0.1] |
| L2 Regularization | - | - | - | - | [1,5,10] |
| Min child weight | - | - | - | [1,3] | - |

RF: Random Forest; LGBM: Light Gradient Boosting Model; XGBM: eXtreme Gradient Boosting Model
